# Activation of BK_Ca_ Channels in Rat Cerebrovascular Smooth Muscle Cells and Vasodilation Induced by Neurogenic H_2_S and Its Relationship with VEGFR_2_

**DOI:** 10.3390/cimb48030284

**Published:** 2026-03-06

**Authors:** Shan Wang, Yu Jiang, Jia-Rong Jiang, Shuai Liang, Ji-Yue Wen, Zhi-Wu Chen, Shuo Chen

**Affiliations:** 1 School of Pharmaceutical Sciences, Anhui Medical University, Hefei 230032, China; 2345010138@stu.ahmu.edu.cn (S.W.);; 2 The Experimental Research Center, Anhui University of Chinese Medicine, Hefei 230038, China

**Keywords:** cystathionine-β-synthase, cerebrovasodilation, neurogenic H_2_S, large-conductance calcium-activated potassium channel, vascular endothelial growth factor receptor

## Abstract

To explore the mechanism of action of CBS-derived H_2_S in inducing cerebral vasodilation and activating BK_Ca_ channels. Sprague–Dawley (SD) rat middle cerebral arteries (MCA) were isolated from rat brains, and a pressure myography system was used to measure the effects of different concentrations of L-cysteine (L-Cys, 1 × 10^−5.5^ to 1 × 10^−3.5^ mol/L), a substrate for cystathionine-β-synthase (CBS)—a hydrogen sulfide (H_2_S)-producing enzyme. Additionally, the effects of pretreatment with the CBS inhibitor amino-oxoacetate (AOAA, 1 mmol/L), the vascular endothelial growth factor receptor 2 inhibitor semaxanib (SU5416, 10 μmol/L), and the large-conductance calcium-activated potassium (BK_Ca_) channel blocker iberiotoxin (IBTX, 100 nmol/L) were investigated to determine their impacts on CBS-derived H_2_S-induced vasodilation. Acute digestion of rat vascular smooth muscle cells (VSMCs) was performed, and whole-cell patch-clamp techniques were used to measure current changes in neurons or astrocytes (ASTs), as well as acutely digested VSMCs, in the presence of L-Cys, AOAA (1 mmol/L), SU5416 (10 μmol/L), and IBTX (100 nmol/L). Additionally, neurons or ASTs were co-cultured with VSMCs to determine CBS-derived H_2_S levels. Neurons or ASTs co-incubated with blood vessels and then treated with L-Cys produced H_2_S, which exhibited a concentration-dependent dilatory effect on middle cerebral artery occlusion (MCA) pre-contracted with 100 nmol/L U46619 (*p* < 0.01). However, the addition of AOAA significantly attenuated this dilatory effect (*p* < 0.01). SU5416 and IBTX significantly inhibited cerebral vascular dilation (*p* < 0.01). H_2_S produced by adding L-Cys after co-incubation of neurons or ASTs with VSMCs significantly increased BK_Ca_ channel current (*p* < 0.01). However, this effect was significantly attenuated after adding AOAA (*p* < 0.01). SU5416 and IBTX significantly inhibited the activation of BK_Ca_ channels (*p* < 0.01). Wild-type rat neurons or astrocytes (ASTs) were co-cultured with CSE(Cystathionine γ-lyase)-knockout vascular smooth muscle cells (VSMCs-CSE KO); the addition of L-Cys significantly increased hydrogen sulfide (H_2_S) levels in the co-culture system (*p* < 0.01), while the addition of AOAA reduced H_2_S production (*p* < 0.01). However, the addition of SU5416 had no statistical significance. Neurogenic H_2_S, the H_2_S produced by neurons and ASTs, could induce cerebral vasodilation in rats via VEGFR_2_(Vascular Endothelial Growth Factor Receptor 2)-mediated activation of BK_Ca_ channels in the smooth muscle cells.

## 1. Introduction

Ischemic stroke is one of the leading causes of morbidity and mortality worldwide. Despite the serious risks, effective treatment options remain limited, necessitating ongoing research and development. Effective treatment of ischemic stroke depends on the functional vascular system, and cerebral vascular tone is regulated by vascular receptors [1,2]. Secondly, activation of the high-conductance calcium-activated potassium channel (BK_Ca_) can also enhance vasodilation [3]. Therefore, studying the regulatory mechanisms of cerebral vascular tension is of great significance for the treatment of ischemic stroke!

The physiological functions of hydrogen sulfide (H_2_S) can be mediated by different ion channels and signaling proteins. Altered metabolism can lead to pathological disorders in some cardiovascular diseases and can regulate inflammation, vascular tension, vascular permeability, mitochondrial function, etc. [4]. Levels in the brain are relatively high. Neurogenic H_2_S in the brain is primarily produced by the action of the L-cysteine pyridoxal 5-phosphate-dependent enzyme—cysteine sulfoxylase (CBS)—in mammals. L-cysteine (L-Cys) is the main substrate for this enzyme [5,6,7,8,9,10]. Combined with other research results, it has been proven that the endogenous H_2_S level in the brains of CBS gene–knockout mice is below the detectable range, indicating that CBS is the main H_2_S-producing enzyme in the brain. CBS is mainly found in the central nervous system, is highly expressed in the hippocampus of the brain, and is primarily located in neurons and astrocytes in the central nervous system [8,11]. This experiment mainly uses neurogenic H_2_S produced by these two types of cells to conduct a series of research projects.

In the middle cerebral artery (MCA) of rats, endogenous H_2_S can activate calcium-activated potassium channels (K_Ca_) in vascular smooth muscle through the action of Ca^2+^ in the vascular system, thereby causing vasodilation [12]. When VSMCs depolarize and contract, Ca^2+^ channels on the cell membrane open, allowing Ca^2+^ to flow into the cell and increase intracellular Ca^2+^ concentration, thereby activating BKCa channels, causing the cell membrane to hyperpolarize, and leading to VSMC relaxation [13]. Our previous studies have confirmed that endothelium-derived H_2_S mediates hyperpolarization of rat vascular smooth muscle cells (VSMCs) and vasodilation of rat basilar artery (BA) and rat middle cerebral artery (MCA) [14,15]. K_Ca_ channels are divided into three subtypes: large conductance (BK_Ca_), medium conductance (IK_Ca_), and small conductance (SK_Ca_; SK1, SK2, and SK3) K_Ca_ channels. BK_Ca_ channels are primarily expressed in vascular smooth muscle cells, and activation of BK_Ca_ channels at this location can lead to vasodilation [16]. The opening of BK_Ca_ channels causes a large outflow of K^+^, leading to hyperpolarization of the cell membrane and having a profound effect on the physiological functions of the cell [17]. To this end, this study aims to investigate how neurogenic H_2_S produced by CBS activates BK_Ca_ channels to dilate cerebral blood vessels.

Vascular endothelial growth factor receptor 2 (VEGFR_2_) plays a crucial role in vascular formation, growth, and branching and is an important target for therapeutic strategies promoting angiogenesis and treating vascular diseases [18]. Ishida et al. confirmed the presence of functional VEGFR_2_ receptors on VSMCs, and these findings also suggest that vascular endothelial growth factor may be involved in regulating VSMC responses in vivo [19]. In addition, Chen et al. found that H_2_S-mediated cerebral vasodilation acts through VEGFR_2_ [20]. Based on this, we aim to investigate whether CBS-generated neurogenic H_2_S also dilates cerebral blood vessels by activating BK_Ca_ channels through the VEGFR_2_ mechanism.

## 2. Materials and Methods

### 2.1. Reagents and Drugs

Acetylcholine (ACh, Lot No. 064K1209), sodium hydrosulfide (NaHS, catalog number: 161527), phenylephrine (PPG, Lot No. 06327DJ), ibrutinib (IBTX, Lot No. 06305DJ), L-cysteine (L-Cys, 52-90-4), Semaxinib (SU5416, Lot No. HY-10347-50mg), aminooxyacetic acid (AOAA, Lot No.C13408), and 9,11-dideoxy-11α,9α-epoxy-methane prostaglandin F2αU46619 (Lot No. 24894192) were purchased from Sigma Chemical Company (St. Louis, MO, USA); Anti-α-smooth muscle actin (anti-α-SMA, Lot No. AF6032) antibody, microtubule-associated protein 2 (MAP-2, Lot No. AF4081) antibody, and glial fibrillary acidic protein (GFAP, Lot No. BF0345) antibody were purchased from Affinity Biosciences (Changzhou, China); A hydrogen sulphide (H_2_S) detection kit was purchased from Nanjing Jiancheng Bioengineering Research Institute (Lot No. 20231228, Nanjing, China). The composition of phosphate-buffered saline (PSS) is as follows (mmol/L): NaCl 118, KCl 4.7, CaCl_2_ 1.6, KH_2_PO_4_ 1.2, MgSO_4_ 1.2, NaHCO_3_ 25, and glucose 11.1. The solution is equilibrated with a mixture of 95% oxygen and 5% carbon dioxide. The composition of the calcium-free buffer solution (calcium-free PSS) is as follows (mmol/L): NaCl 50, sodium glutamate 70, KCl 5.6, MgCl_2_ 2, HEPES 10, D(+)-Glucose 10. The pH of the calcium-free PSS was adjusted to 7.2 using 5% NaOH, then aliquoted through a 0.22 μm microporous filter membrane and stored at −20 °C. The composition of the BKCa channel cell bath solution is as follows (mmol/L): NaCl 140, KCl 5, CaCl_2_ 2, MgCl_2_ 1.2, HEPES 10, and D(+)-Glucose 10. The pH is adjusted to 7.2 using 5% NaOH, then filtered through a 0.22 μm microporous membrane, and stored at −20 °C. BKCa channel electrode filling solution (mmol/L): K-glutamine 105, KCl 35, MgCl_2_ 1, CaCl_2_ 2.1, Na_2_ATP 5, EGTA 5.1, and HEPES 10. The pH was adjusted to 7.35–7.45 using 5% KOH, then the solution was aliquoted through a 0.22 μm microporous filter membrane and stored at −20 °C. Cell digestion enzymes: Weigh 0.01 g of collagenase II, 0.046 g of papain, 0.024 g of bovine serum albumin (BSA), and 0.0088 g of dithiothreitol (DTT) and place into a 5 mL centrifuge tube, prepare with calcium-free PSS, then aliquot and store at −20 °C.

### 2.2. Animals

Neonatal Sprague–Dawley (SD) rats within 24 h and adult SD rats were bought from the Experimental Animal Center of Anhui Medical University. Wild-type (WT), CSE-knockout (CSE KO) mice were obtained from Shanghai Biomodel Organism Science and Technology Development Co., Ltd. (Shanghai, China). All animals were housed in the Animal Center of Anhui Medical University, where they had unrestricted access to food and water. The environmental conditions for raising the animals were maintained at a humidity level of 54 ± 2% and a temperature of 22 ± 2 °C. The experiments received approval from the Ethical Committee of Anhui Medical University, and all experimental protocols adhered to the regulations established by the Animal Care and Use Committee at Anhui Medical University. These protocols comply with the guidelines outlined in the “Guide for the Care and Use of Laboratory Animals,” published by the US National Institutes of Health (NIH Publication No. 85-23, revised 2011). In the subsequent descriptions of cell isolation and culture in Section 2.4, Section 2.5 and Section 2.8, all animals were anesthetized prior to decapitation. Supplementary information on the experimental methods for these animals is provided in Appendix A.

### 2.3. Pressure Myography

Changes in the diameter of extracorporeal vessels were recorded using pressure myography, as described above [14]. SD rats were anesthetized with pentobarbital (50 mg/kg) administered intraperitoneally, decapitated, and the brain tissue was quickly removed and placed in pre-cooled, oxygenated physiological saline solution (PSS), then cut into 3–5 mm long vascular segments. Carefully secure both ends of the vascular loop to the pre-adjusted double-ended glass micropipettes within the chamber of the myography system. Install the system into the perfusion chamber of the DMT-114P pressure myography system (Danish Myo Technology, Aarhus, Denmark) and fill it with 37 °C PSS containing 95% O_2_ and 5% CO_2_. The vascular lumen was also perfused with the same oxygenated and 37 °C PSS. The chamber was then placed on the microscope stage connected to the digital imaging system, and pressure not exceeding 120 mmHg was applied to the vascular ring using a glass micropipette. The arterial segment will be fixed in the perfusion chamber of the DMT-114P pressure electromyography system for 1 h, and then U46619 will be added to the arterial segment lumen perfusion solution at a final concentration of 100 nmol/L to induce stable vasoconstriction. Once sustained vasoconstriction was established approximately 1 min after administration of the U46619 drug, the dilatory response of the MCA to the relevant experimental group drugs was measured to investigate the mechanism of action of H_2_S in dilating blood vessels and its relationship with VEGFR_2_.

The experiment was divided into the following groups: PSS group, ACh group, L-Cys group, AOAA group, Neurons group, ASTs group, SU5416 group, IBTX group, Neurons+L-Cys group, ASTs+L-Cys group, Neurons+L-Cys+AOAA, ASTs+L-Cys+AOAA group, Neurons+L-Cys+SU5416 group, ASTs+L-Cys+SU5416 group, Neurons+L-Cys+IBTX group, and ASTs+L-Cys+IBTX group.

The tube diameter is continuously monitored by the pressure myography system software, and the degree of vasodilation is expressed as a percentage of the maximum diameter. Based on changes in the vessel diameter, the calculation formula is as follows:

Vasodilation (%) = (Dx − Dmin)/(Dmax − Dmin) × 100%

where Dx is the diameter after administration, Dmin is the stable diameter after the addition of U46619 (pre-constriction), and Dmax is the initial diameter obtained after 60 min of vascular equilibrium.

### 2.4. Isolation and Culture of Rat Basilar Artery Vascular Smooth Muscle Cells (VSMCs)

Referring to the previous literature on the preparation of primary cultured rat cerebral basilar artery (CBA) VSMCs [21,22,23], we isolated and cultured rat CBA VSMCs. Healthy adult SD rats were anesthetized with pentobarbital (50 mg/kg) via intraperitoneal injection, and their heads were quickly removed. The brains were then placed in pre-cooled sterile phosphate-buffered saline (PBS, pH 7.4). Tissue blocks were resuspended in DMEM/F12 medium containing 20% fetal bovine serum (FBS), transferred to cell culture flasks, and a pipette tip was used to spread the tissue blocks evenly across the bottom of the flasks. After inverting the 37 °C incubator for 2–4 h, the culture flask was flipped so that the culture medium covered the tissue block, and continued to incubate in the incubator. Cells from the 3rd to 5th generations were used for subsequent experiments.

### 2.5. Isolation and Culture of Rat Hippocampal Neurons

Primary cultured hippocampal neurons from newborn Sprague–Dawley rats were prepared according to previous literature [24]. The hippocampus tissue was then rapidly separated and removed from the brain and cut into small pieces in PBS (pH 7.45). After digesting with 0.125% trypsin solution at 37 °C for 20 min, the cells were collected and resuspended in DMEM/F12 (1:1) containing 20% FBS and 1% penicillin–streptomycin. Subsequently, the dissociated cells were seeded into culture flasks pre-coated with poly-L-lysine (1 mg·mL^−1^). The cells were then incubated at 37 °C, 95% O_2_/5% CO_2_. After 24 h, half of the medium was replaced with Neurobasal™ medium (Gibco Invitrogen, Carlsbad, CA, USA) containing 2% B27, 0.5 mM L-glutamine, and 1% penicillin–streptomycin solution. The medium was replaced with fresh medium every 2–3 days. The cells were ready to be used for experiments after 8–10 days of culture.

### 2.6. Isolation and Culture of Rat Astrocytes (ASTs)

Primary cultured astrocytes from newborn SD rats were prepared according to previous literature [25,26]. In short, the brains of 1–3-day-old SD rats were removed from the ice plate, and the cerebral cortical tissues were routinely isolated. The soft meninges were peeled off, and the tissue was homogenized using a pipette to prepare a cell suspension. The suspension was filtered through a 200-mesh cell strainer, and the filtrate was centrifuged at 300× *g* for 5 min. The supernatant was discarded, and the cell suspension was resuspended in DMEM/F12 medium containing 10% fetal bovine serum. After seeding and culture, oligodendrocytes and microglia were removed by shaking on a rocking bed. After primary culture for 8–12 days, the cells were passaged. The second-generation cells were identified by immunofluorescence staining with an anti-GFAP antibody.

### 2.7. Immunofluorescence Staining

Immunofluorescence identification of primary cultured vascular smooth muscle cells (VSMCs), neurons, and astrocytes (ASTs) was performed according to previous reports [27,28]. The brief steps are as follows: Once cell confluence reaches 80%, wash the cells with sterile phosphate-buffered saline (PBS). First, fix the cells with 4% paraformaldehyde for 15 min, then permeabilise them with 0.1% Triton X-100, followed by blocking with 10% normal goat serum for 1 h. Next, add the primary antibody diluted 1:300 (microtubule-associated protein 2 antibody for neuronal cells, glial fibrillary acidic protein antibody for astrocytes, and anti-α-smooth muscle actin antibody for vascular smooth muscle cells), and incubate overnight at 4 °C. The next day, add fluorescently labeled secondary antibody in the dark and stain the cell nuclei with 4′,6-diaminophenylindole (DAPI). Finally, observe the immunofluorescence staining results under a fluorescence inverted microscope or a laser scanning confocal microscope.

### 2.8. Acute Separation of VSMC and CBA

VSMCs were isolated from the basilar artery of wild-type rats according to the improved and optimized steps described in previous studies [29]. In summary, SD rats were selected, and after decapitation, the procedure was performed on ice to isolate the middle cerebral artery and thoroughly remove the peripheral tissue attached to the vessel. The brain was placed in 0.5 mL of cell digestion solution (a calcium-free PSS containing type II collagenase and papain) and cut into small pieces with scissors (catalog number: 6801-3, total length: approximately 80–100 mm, blade length: approximately 10–20 mm). The tissue was digested in a 37 °C water bath for 30–40 min. Then, the supernatant was removed, and 1 mL of BSA solution was added to the centrifuge tube. It was then left to stand on ice for 10 min before the supernatant was removed. Finally, the sample was washed 2–3 times with 1 mL of calcium-free PSS buffer, the supernatant was removed, and 0.5 mL of calcium-free PSS was added, and the sample was incubated at 4 °C for 1–2 h. To ensure cell viability, the experiment was completed within 2 h.

### 2.9. Whole-Cell Patch Clamp Recording

Whole-cell patch-clamp recordings of VSMCs were performed as described in previous studies [30]. VSMCs were placed in the perfusion chamber on the microscope stage, and 2 mL of BKCa cell bath solution was added. The channel was recorded under voltage clamp conditions. Patch pipettes were prepared using a micro-pipette puller (P97, Sutter, Union City, CA, USA) with a resistance of 2–5 MΩ, and then the BK_Ca_ channel electrode filling solution was added. The BK_Ca_ channel current in VSMCs was recorded for 500 ms under whole-cell voltage clamp mode with a clamp voltage of −60 mV and a step square wave pulse from −60 mV to +90 mV in 10 mV steps. All drugs were dissolved in the extracellular solution and used only after steady-state conditions were achieved, which were determined by the current maintaining a constant amplitude. Whole-cell currents were recorded using an Axopatch 700B amplifier (Axon Instruments, Union City, CA, USA). Physiological signals were acquired and analyzed using pClamp 10.5 software (Axon Instruments, USA). To account for differences in cell size among target cells, the current was normalized by dividing it by cell capacitance, resulting in current density. All experiments were conducted at room temperature (20–26 °C).

### 2.10. Two-Cell Co-Culture Model

Neuronal cells–vascular smooth muscle cells (neurons–VSMCs)/hippocampal neuronal astrocytes–vascular smooth muscle (astrocyte–VSMCs) co-culture model construction: Primary cultured brain vascular smooth muscle CSE–knockout cells (CSE-KO VSMCs, 1 × 10^6^ cells) were seeded into the lower chamber of a Transwell, and primary cultured neuronal cells or hippocampal astrocytes (neurons or ASTs, 1 × 10^5^ cells) were seeded into the upper chamber. DMEM/F12 cell culture medium containing 10% fetal bovine serum (FBS) was added to each well and incubated at 37 °C in a 5% CO_2_ cell culture incubator.

### 2.11. H_2_S Measurement

Cell culture media were collected after administration of the above experiments. H_2_S was detected at 450 nm using H_2_S assay kits based on the formation of methylene blue as previously described [30] according to the manufacturer’s instructions.

### 2.12. Statistical Analysis

The data were expressed as means ± standard deviation (x ± s). All statistical analyses were conducted using GraphPad Prism version 10.1 software (GraphPad Software, San Diego, CA, USA). Intergroup comparisons were performed using one-way analysis of variance (ANOVA), with a value of *p* < 0.05 considered statistically significant.

## 3. Results

### 3.1. Identification of Primary Cultured Rat Cerebrovascular VSMCs, Hippocampal Neurons, and ASTs

**Figure 1:**
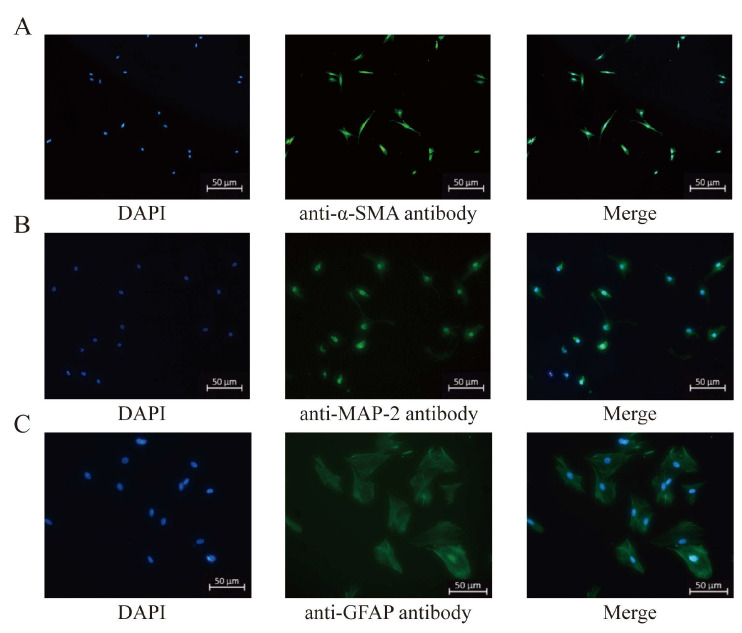
Identification of primary cultured rat cerebral basilar artery vascular smooth muscle cells, hippocampal neurons, and astrocytes (Immunofluorescence examination, ×50). (**A**): Rat cerebrovascular smooth muscle cells. α-SMA, the marker of vascular smooth muscle cells, was stained green by α-SMA antibody and presented in the cell bodies. (**B**): Rat hippocampal neurons. MAP-2, the marker of neurons, was stained green by MAP-2 antibody, and expressed in cell bodies and axons. (**C**): Rat astrocytes. The marker of astrocytes, GFAP, was stained green by the GFAP antibody and presented in cell bodies. The nuclei of these three kinds of cells were stained blue by DAPI.

An immunofluorescence assay was used for identifications of primary cultured VSMCs, neurons, and ASTs. As shown in Figure 1A, the nuclei of the cultured rat cerebrovascular cells were dyed blue by DAPI, and the cell bodies were stained green by the antibody against the marker of VSMCs, α-SMA, confirming that the cultured rat cerebrovascular cells were ECs.

Figure 1B showed that the nuclei of the cultured rat hippocampal cells were dyed blue by DAPI, and the cell bodies and axons were stained green by the antibody against neuronal marker, α-SMA, demonstrating that the cultured rat hippocampal cells were neurons.

Figure 1C that the nuclei of the cultured rat hippocampal cells were stained blue by DAPI, and the cell bodies were stained green by the antibody against GFAP, the marker of ASTs. Therefore, the cultured cells were identified as rat ASTs.

### 3.2. H_2_S Production Increased by L-Cys via Neurogenic CBS in the H/R-Treated Co-Culture of Nerve Cells with VSMCs

CBS 2. S, with CBS mainly located in nerve cells and CSE primarily present in VSMCs. To observe the effect of neurogenic H_2_S on VSMCs, we co-cultured rat nerve cells (neurons or ASTs) with the cerebral artery VSMCs from CSE knockout mice, and measured H_2_S levels in the co-culture.

**Figure 2:**
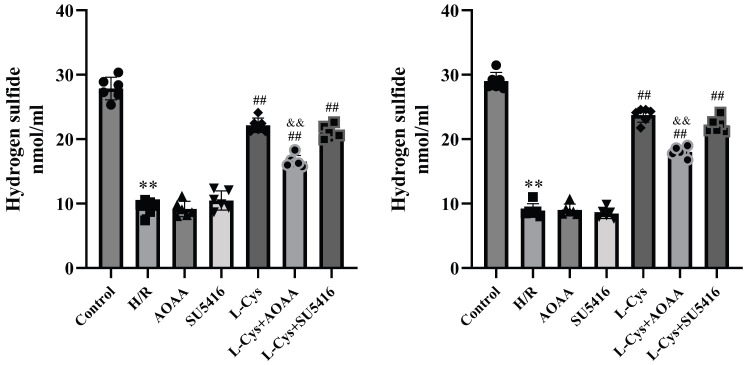
Effects of H_2_S synthase substrate L-Cys and inhibitor AOAA and VEGFR_2_ blocker SU5416 on H_2_S production in the H/R-treated co-culture of rat cerebrovascular vascular smooth muscle cells with nerve cells (H_2_S Measurement, Mean ± SD, n = 6). (**A**): In the H/R-treated rat cerebrovascular smooth muscle cells with hippocampal neurons. (**B**): In the H/R-treated rat cerebrovascular smooth muscle cells with astrocytes. Compared with the Control group, ** *p* < 0.01; compared with the H/R group, ^##^ *p* < 0.01; compared with the L-Cys group, ^&&^ *p* < 0.01.

Figure 2A,B showed that in the neurons–VSMCs–CSE–KO co-culture or the ASTs-VSMCs-CSE-KO co-culture, the H_2_S level in the H/R group reduced significantly compared with that in the control group (*p* < 0.05), L-Cys markedly inhibited the reduction in H_2_S level by H/R (*p* < 0.05); The CBS inhibitor AOAA or VEGFR_2_ blocker SU5416 treatment alone had no significant effect on the reduction in H_2_S level by H/R (*p* ˃ 0.05), AOAA but not SU5416 remarkably attenuated the inhibition of L-Cys on the H/R-induced the reduction in H_2_S level (*p* < 0.05). The results suggested that L-Cys could increase H_2_S production in the co-culture treated with H/R via the CBS in neurons or ASTs.

### 3.3. Mediation of Rat Neuronal CBS in L-Cys-Induced Vasorelaxation in Rat MCA

**Figure 3:**
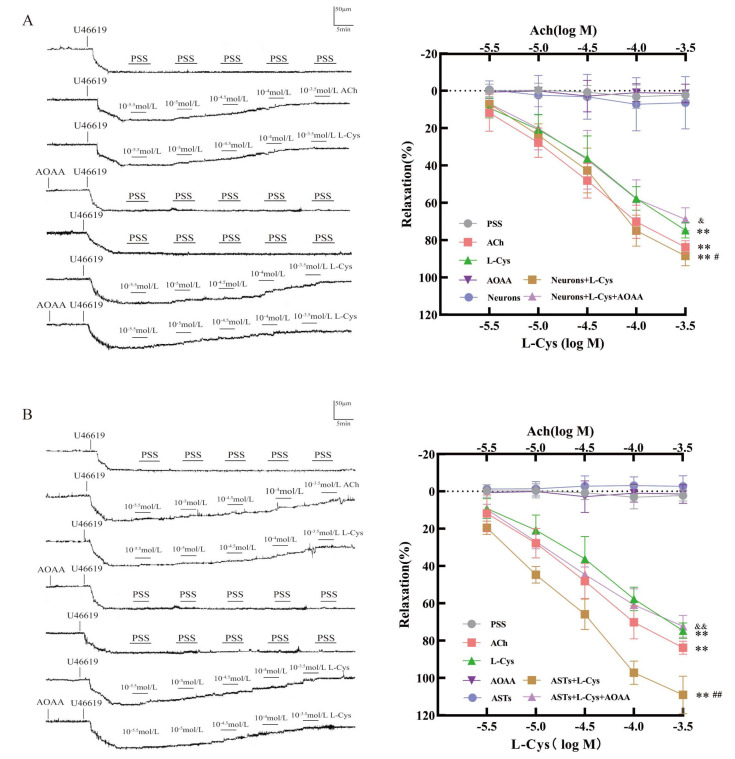
Effect of H_2_S synthase inhibitor AOAA on the L-Cys-induced vasorelaxation of rat MCA mediated by rat neurons and ASTs (pressure myography, mean ± SD, n = 5). (**A**): The vasodilation mediated by rat hippocampal neurons. (**B**): The vasodilation mediated by rat ASTs. Neurons: 1 × 10^5^ cells/mL; ASTs: 1 × 10^5^ cells/mL; AOAA: 1 mmol/L. Compared with the Control group, ** *p* < 0.01; compared with the L-Cys group, ^#^ *p* < 0.05, ^##^ *p* < 0.01; compared with the Neurons+L-Cys group, ^&^ *p* < 0.05, ^&&^ *p* < 0.01.

As shown in Figure 3A, in a range of 1 × 10^−5.5^~1 × 10^−3.5^ mol/L, L-Cys dramatically relaxed the rat MCA pre-contracted by U46619 (100 nmol/L) compared with the PSS group (*p* < 0.01). ACh (1 × 10^−5.5^ ~ 1 × 10^−3.5^ mol/L) had a similar vasodilatory effect in the MCA (*p* < 0.01). Rat hippocampal neurons (1 × 10^5^ cells/mL) had no obvious effect in the MCA (*p* ˃ 0.05), but significantly enhanced the vasodilation of L-Cys in the MCA (*p* < 0.05). The results suggested that L-Cys not only directly relaxed rat MCA but also induced dilation of rat MCA through rat hippocampal neurons.

Figure 3A also indicated that AOAA (1 mmol/L) did not dilate rat MCA, but it remarkably attenuated the enhanced effect of the neurons on L-Cys-induced vasorelaxation in rat MCA (*p* < 0.05). These results demonstrate that CBS in rat neurons mediates L-Cys-induced vasorelaxation of the rat MCA.

### 3.4. Mediation of the CBS in Rat ASTs in L-Cys-Induced Vasorelaxation in Rat MCA

As shown in Figure 3B, rat ASTs (1 × 10^5^ cells/mL) did not obviously induce dilation in the MCA (*p* ˃ 0.05), but remarkably increased the vasodilation of rat MCA to L-Cys (*p* < 0.05). The results suggested that ASTs mediated the vasodilation of L-Cys in rat MCA.

Figure 3B further indicated that AOAA (1 mmol/L) markedly attenuated the increased effect of the ASTs on L-Cys-induced vasorelaxation in rat MCA (*p* < 0.05). These results indicate that CBS in rat ASTs could also mediate L-Cys-induced vasorelaxation of the rat MCA.

### 3.5. Effect of SU5416 on L-Cys-Induced Relaxation of Rat MCA Mediated by the CBS in Rat Nerve Cells

**Figure 4:**
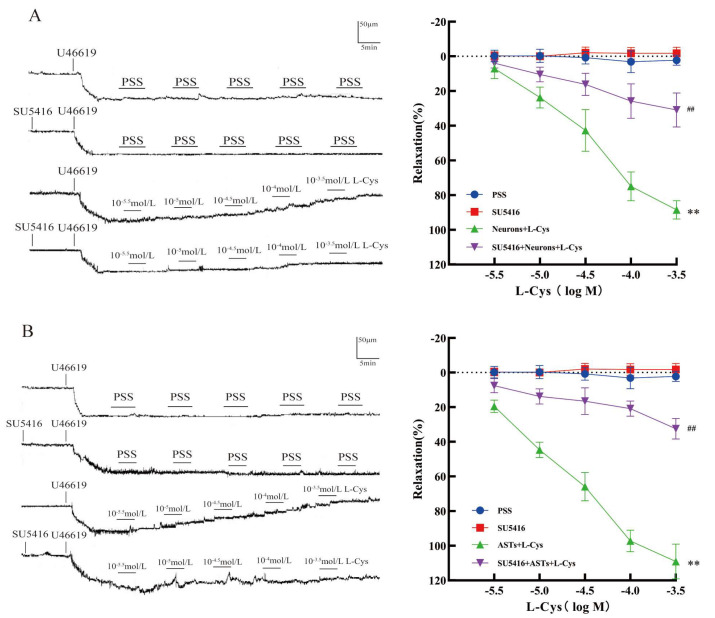
Effect of VEGFR_2_ blocker SU5416 on the L-Cys-induced relaxation of rat MCA mediated by rat nerve cells (Pressure myography, mean ± SD, n = 5). (**A**): The vasodilation mediated by rat hippocampal neurons. (**B**): The vasodilation mediated by rat ASTs. Neurons: 1 × 10^5^ cells/mL; ASTs: 1 × 10^5^ cells/mL; SU5416: 100 nmol/L. Compared with the Control group, ** *p* < 0.01; compared with the Neurons+L-Cys group, ^##^ *p* < 0.01; compared with the ASTs+L-Cys group, ^##^ *p* < 0.01.

As shown in Figure 4A, VEGFR_2_ blocker SU5416 (10 μmol/L) did not dilate rat MCA, but it remarkably attenuated the enhanced effect of the neurons on L-Cys-induced vasorelaxation in rat MCA (*p* < 0.01). Figure 4B showed that SU5416 also significantly attenuated the enhanced effect of the ASTs on vasodilation of rat MCA induced by L-Cys (*p* < 0.01). The results demonstrated that VEGFR_2_ was involved in the L-Cys-induced relaxation of rat MCA mediated by rat hippocampal neurons and ASTs.

### 3.6. Effect of IBTX on the Relaxation of Rat MCA Induced by the Co-Application of L-Cys and Rat Nerve Cells

**Figure 5:**
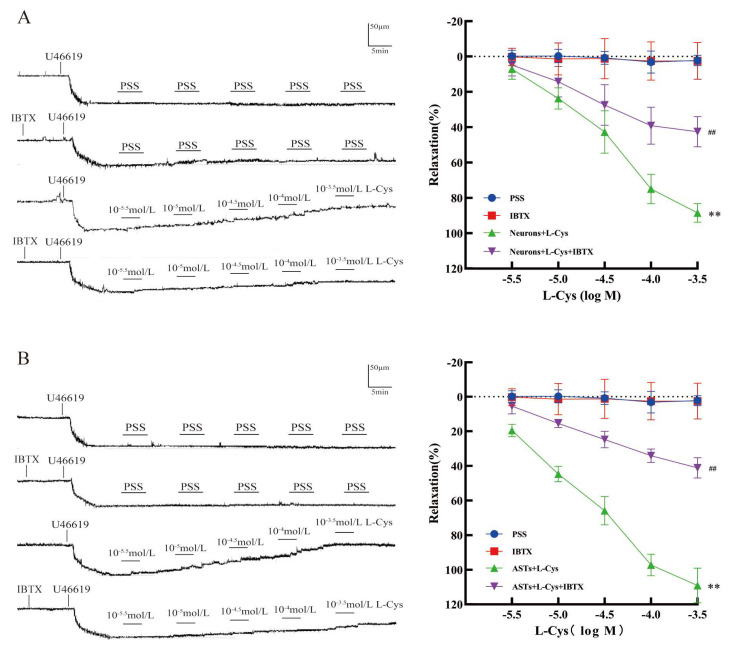
Effect of BK_Ca_ blocker IBTX on the vasodilation of rat MCA induced by co-application of L-Cys and rat nerve cells (Pressure myography, mean ± SD, n = 5). (**A**): The vasodilation induced by the co-application of L-Cys and rat hippocampal neurons. (**B**): The vasodilation induced by co-application of L-Cys and rat ASTs. Neurons: 1 × 10^5^ cells/mL; ASTs: 1 × 10^5^ cells/mL; IBTX: 100 nM. Compared with the Control group, ** *p* < 0.01; compared with the Neurons+L-Cys group, ^##^ *p* < 0.01; compared with the ASTs+L-Cys group, ^##^ *p* < 0.01.

As shown in Figure 5A,B, in rat MCA pre-contracted with 100 nmol/L U46619, compared with the PSS group, the BK_Ca_ blocker IBTX (100 nmol/L) had no obvious relaxation (*p* ˃ 0.05); but it remarkably attenuated vasodilation of rat MCA induced by co-application of L-Cys and rat hippocampal neurons or ASTs (the L-Cys+Neurons group, or the L-Cys+ASTs group) (*p* < 0.01). The result suggested that BK_Ca_ was involved in the L-Cys-induced relaxation of rat MCA mediated by rat hippocampal neurons or ASTs.

### 3.7. Effect of SU5416 on Exogenous H_2_S-Increased Current of BK_Ca_ Channels in Rat MCA VSMCs

The aforementioned result indicated that L-Cys increased the H_2_S production in the H/R-treated co-culture via the neurogenic CBS, together with attenuation of SU5416 on the vasodilation of L-Cys mediated by neurons or ASTs. Our results suggested that an involvement of VEGFR_2_ in the vasodilation mediated by the neurogenic CBS-produced H_2_S. Thus, the role of VEGFR_2_ in H_2_S-increased BK_Ca_ current was examined.

**Figure 6:**
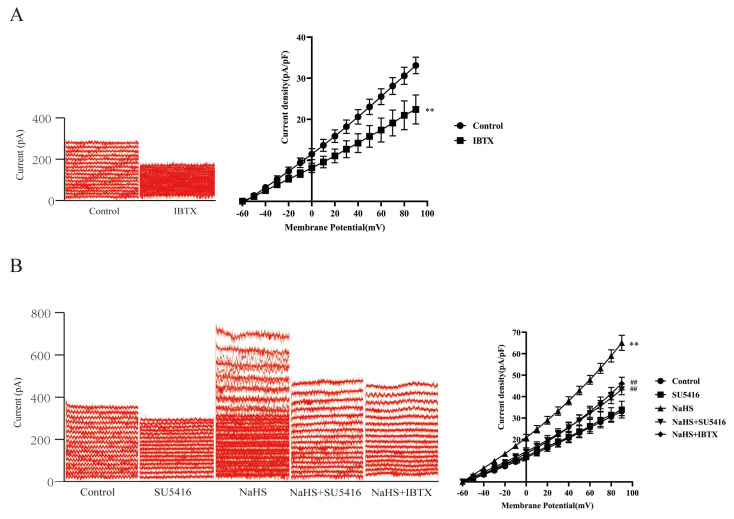
NaHS increased the current of BK_Ca_ channels in rat CBA vascular smooth muscle cells, and the effects of BK_Ca_ channel blocker IBTX and VEGFR_2_ blocker SU5416 on the increased current (whole-cell patch clamp recording, mean ± SD, n = 5). (**A**): Current characteristics of BK_Ca_ channels. Left panel: original traces of BK_Ca_ current under control conditions and application of IBTX; right panel: curves of current density-voltage relationship. (**B**): NaHS increased the BK_Ca_ current, and the effects of IBTX and SU5416 on the BK_Ca_ current. Left panel: original traces of BK_Ca_ current under various treatments; right panel: curves of current density-voltage relationship. L-Cys: 100 μmol/L; IBTX: 100 nM; SU5416: 100 μmol/L; NaHS: 100 μmol/L. Compared with the Control group, ** *p* < 0.01; compared with the NaHS group, ^##^ *p* < 0.01.

BK_Ca_ channel currents were recorded using the whole-cell patch-clamp technique, with a holding voltage ranging from −60 mV to +90 mV. The stimulation protocol consisted of step voltage stimulation, with 10 mV steps lasting 500 ms each. As shown in Figure 6A, a noise-like outward current whose amplitude increased significantly with increasing holding voltage, consistent with the kinetic characteristics of BK_Ca_ channel currents. And this outward current was substantially inhibited by the BK_Ca_ channel blocker IBTX (100 nmol/L) (*p* < 0.01), confirming that the evoked current in the VSMCs was a BK_Ca_ channel current. Figure 6B indicated that H_2_S donor NaHS (100 μmol/L) considerably increased the noise-like outward current in the VSMCs, which was markedly attenuated by IBTX (*p* < 0.01), suggesting that exogenous H_2_S could increase the current of BK_Ca_ channels in the VSMCs.

Figure 6B also indicated that VEGFR_2_ blocker SU5416 (10 μmol/L) did not affect the current of BK_Ca_ channels evoked in the VSMCs (*p* ˃ 0.05), but dramatically attenuated the NaHS-increased current of BK_Ca_ channels (*p* < 0.01), demonstrating that VEGFR_2_ was involved in exogenous H_2_S activating on the BK_Ca_ channels in rat cerebral VSMCs.

### 3.8. Effect of CSE Inhibitor PPG on L-Cys-Increased Current of BK_Ca_ Channels in Rat MCA VSMCs

**Figure 7:**
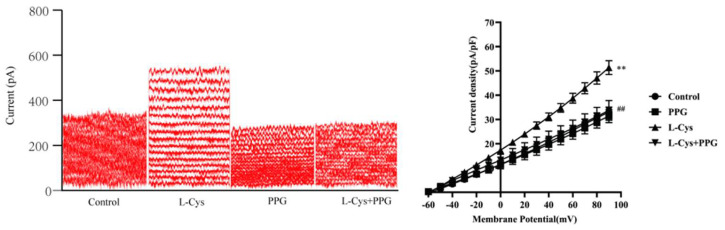
L-Cys increased the current of BK_Ca_ channels in rat CBA vascular smooth muscle cells (Whole-cell patch clamp recording, mean ± SD, n = 5). **Left** panel: original traces of BK_Ca_ current under control conditions and application of L-Cys, PPG, and L-Cys + PPG; **right** panel: curves of current density–voltage relationship. L-Cys: 100 μmol/L; PPG: 100 nM. Compared with the Control group, ** *p* < 0.01; compared with the L-Cys group, ^##^ *p* < 0.01.

As shown in Figure 7 compared with the control group, L-Cys significantly increased the current of BK_Ca_ channels in rat MCA VSMCs (*p* < 0.01). Since PPG is a specific inhibitor of the endogenous H_2_S-synthesizing enzyme cystathionine γ-lyase (CSE), which was dramatically inhibited by the H2S-producing enzyme CSE inhibitor PPG (*p* < 0.01), these results suggest that L-Cys may regulate BK_Ca_ channel activity by activating CSE to produce endogenous H_2_S.

### 3.9. The Current of BK_Ca_ Channels in Rat MCA VSMCs Mediated by the CBS in Rat Neurons or ASTs

**Figure 8:**
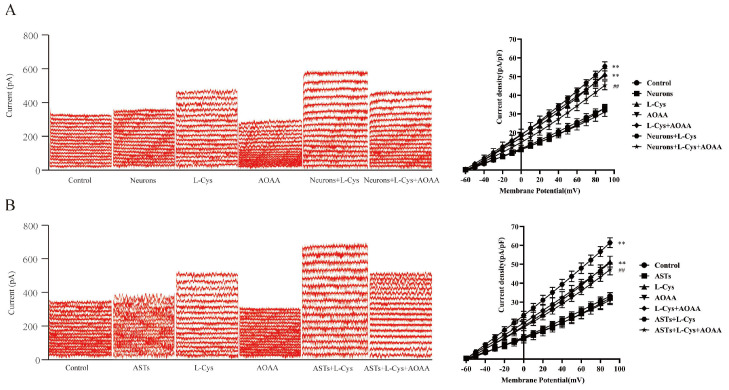
The CBS in rat neurons or ASTs mediates L-Cys-induced BK_Ca_ channel current changes in rat MCA VSMCs (Whole-cell patch clamp recording, mean ± SD, n = 5). (**A**): neurons mediate L-Cys-induced BK_Ca_ channel current changes in rat MCA VSMCs. Left panel: original traces of neurons mediating L-Cys-induced BK_Ca_ channel current under various treatments; right panel: curves of current density-voltage relationship. (**B**): ASTs mediate L-Cys-induced BKCa channel current changes in rat MCA VSMCs. Left panel: original traces of ASTs mediating L-Cys-induced BK_Ca_ channel current under various treatments; right panel: curves of current density-voltage relationship.Neurons: 2 × 10^5^ cells/mL; ASTs: 2 × 10^5^ cells/mL. L-Cys: 100 μmol/L; AOAA: 1 mmol/L. Compared with the Control group, ** *p* < 0.01; compared with the neurons+L-Cys group, ^##^ *p* < 0.01; compared with the ASTs+L-Cys group, ^##^ *p* < 0.01.

Figure 8A,B showed that compared with the control group, treatment with rat neurons or ASTs alone did not significantly affect the current of BK_Ca_ channels in rat MCA VSMCs (P ˃ 0.05); however, co-application of L-Cys with rat neurons or ASTs substantially increased the current of BK_Ca_ channels in the VSMCs compared with the L-Cys group, the Neurons group, or the ASTs group (*p* < 0.01), suggesting that L-Cys could also activate BK_Ca_ channels in rat MCA VSMCs through neurons or ASTs.

Figure 8A,B also indicated that AOAA had no significant effect on the current of BK_Ca_ channels in rat MCA VSMCs (*p* ˃ 0.05), but it remarkably attenuated the increase in the current of BK_Ca_ channels induced by co-application of L-Cys with rat neurons or ASTs (*p* < 0.01), demonstrating that L-Cys could activate BK_Ca_ channels in rat MCA VSMCs through the CBS in neurons or ASTs.

### 3.10. Involvement of VEGFR_2_ in the Current of BK_Ca_ Channels Increased by L-Cys with Rat Neurons or ASTs

**Figure 9:**
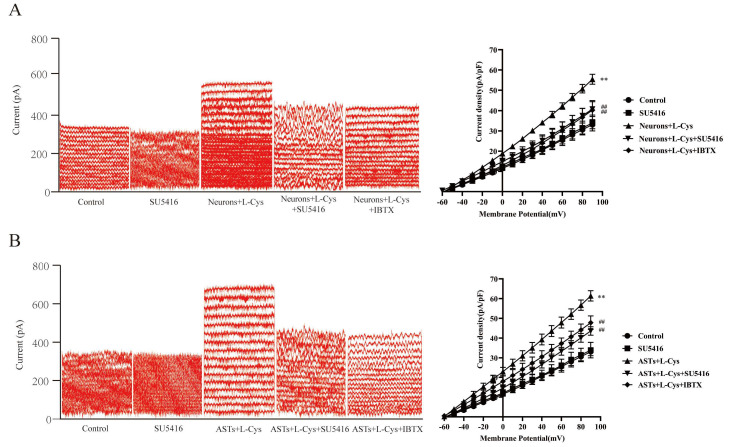
Effect of VEGFR_2_ blocker SU5416 on the L-Cys-induced BK_Ca_ channel current in rat MCA VSMCs mediated by neurons or ASTs (whole-cell patch clamp recording, mean ± SD, n = 5). (**A**): SU5416 on the L-Cys-induced BK_Ca_ channel current in rat MCA VSMCs mediated by neurons. Left panel: original traces of SU5416 on BK_Ca_ channel current in rat MCA VSMCs mediated by neurons under various treatments; right panel: curves of current density-voltage relationship. (**B**): SU5416 on the L-Cys-induced BK_Ca_ channel current in rat MCA VSMCs mediated by ASTs. Left panel: original traces of SU5416 on BK_Ca_ channel current in rat MCA VSMCs mediated by ASTs under various treatments; right panel: curves of current density-voltage relationship. Neurons: 2 × 10^5^ cells/mL; ASTs: 2 × 10^5^ cells/mL. L-Cys: 100 μmol/L; SU5416:100 μmol/L. Compared with the Control group, ** *p* < 0.01; compared with the Neurons+L-Cys group, ^##^ *p* < 0.01; compared with the ASTs+L-Cys group, ^##^ *p* < 0.01.

Figure 9A,B indicated that VEGFR_2_ blocker SU5416 (10 μmol/L) had no effect on the current of BK_Ca_ channels in the VSMCs (*p* ˃ 0.05), but dramatically inhibited the increase in the current of BK_Ca_ channels induced by co-administration of L-Cys with rat neurons or ASTs (*p* < 0.01). IBTX had a similar inhibitory effect on the increase in the current of BK_Ca_ channels induced by L-Cys combined with rat neurons or with ASTs. The results demonstrated that VEGFR_2_ was involved in L-Cys-activated BK_Ca_ channels in rat cerebral VSMCs mediated by neurons or ASTs.

## 4. Discussion

This study demonstrates that neurogenic H_2_S derived from neurons and ASTs can activate BK_Ca_ channels in rat cerebral VSMCs, thereby inducing significant dilation of rat cerebral arteries. Further investigation reveals that this cerebrovascular dilatory effect is one of the mechanisms by which vascular endothelial growth VEGFR_2_ exerts its protective role against ischemic brain injury.

In the co-culture system of CSE-KO rat basilar artery VSMCs with neurons or ASTs, we observed that supplementation of L-Cys—the substrate for endogenous H_2_S synthases—to wild-type neurons or ASTs significantly elevated the H_2_S concentration in the culture supernatant. Moreover, this elevating effect was abrogated by AOAA, a specific inhibitor of endogenous H_2_S synthase; in contrast, the VEGFR_2_ inhibitor SU5416 had no statistically significant impact on H_2_S production (*p* > 0.05).

Vascular reactivity assays showed that L-Cys (at concentrations ranging from 10^−5.5^ to 10^−3.5^ mol/L) exerted a potent vasodilatory effect on rat basilar arteries pre-contracted with U46619. Notably, this dilatory effect was further enhanced when neurons or ASTs were co-administered with the CBS substrate. Pre-incubation with AOAA significantly attenuated the L-Cys-induced vasodilation, indicating that neurogenic H_2_S catalyzed by CBS in neurons or ASTs mediates the dilatory response of rat cerebral vessels. Consistent with this, pre-treatment with SU5416 also markedly reduced the vasodilatory effect, suggesting that CBS-derived H_2_S-induced cerebrovascular dilation is VEGFR_2_-dependent. Additionally, pre-incubation with the BK_Ca_ channel-specific inhibitor IBTX significantly blunted the L-Cys-induced vasodilation, confirming the involvement of BK_Ca_ channels in this process.

Patch-clamp experiments further verified that exogenous H_2_S (donated by NaHS) significantly activated BK_Ca_ channels in VSMCs, while this activating effect was significantly diminished by pre-incubation with SU5416—demonstrating that VEGFR_2_ is involved in exogenous H_2_S-mediated BK_Ca_ channel activation. Similarly, neurogenic H_2_S produced by neurons or ASTs also effectively activated BK_Ca_ channels, and this effect was notably suppressed by SU5416. Collectively, these results confirm that CBS-derived H_2_S activates BK_Ca_ channels in a VEGFR_2_-dependent manner.

In conclusion, this study is the first to confirm that neurogenic CBS-derived H_2_S elicits cerebrovascular dilation in rats by VEGFR_2_-mediated activation of BK_Ca_ channels on cerebral VSMCs. These findings provide novel insights into the mechanism underlying the therapeutic potential of CBS-derived H_2_S in ischemic cerebrovascular diseases, laying a foundation for the development of targeted therapeutic strategies.

## Data Availability

The original contributions presented in this study are included in the article. Further inquiries can be directed to the corresponding authors.

## Abbreviations

The following abbreviations are used in this manuscript:

BK_Ca_	Large-conductance calcium-activated potassium
VEGFR_2_	Vascular endothelial growth factor receptor 2
CBS	Cystathionine-β-synthase
CSE	Cystathionine-γ-lyase
ECs	Endothelial cells
ASTs	Astrocytes
VSMCs	Vascular smooth muscle cells
H/R	Hypoxia/reoxygenation
H_2_S	Hydrogen sulfide
I/R	Ischemia/reperfusion
KO	Knockout
WT	Wild-type

## Appendix A. In Vivo Animal Experimental Methods

### Appendix A.1. Experimental Animals

WT and CSE-KO rat (C57BL/6J background) aged 8–10 weeks (body weight: 20–25 g) and SD rats aged 8–10 weeks (body weight: 200–300 g) with a 1:1 female-to-male ratio were obtained from Shanghai Biomodel Organism Science and Technology Development Co., Ltd. All mice were housed in the Animal Center of Anhui Medical University under controlled environmental conditions (temperature: 22 ± 2 °C; humidity: 54 ± 2%) with free access to food and water. Prior to the experiment, animals were acclimatized to the facility for 7 days.

### Appendix A.2. Experimental Design

Stratified randomization by sex and weight was performed using a computer-generated sequence. WT mice were randomly allocated to the following groups: PSS group, ACh group, L-Cys group, AOAA group, Neurons group, ASTs group, SU5416 group, IBTX group, Neurons+L-Cys group, ASTs+L-Cys group, Neurons+L-Cys+AOAA, ASTs+L-Cys+AOAA group, Neurons+L-Cys+SU5416 group, ASTs+L-Cys+SU5416 group, Neurons+L-Cys+IBTX group, and ASTs+L-Cys+IBTX group. The experimental unit was defined as the individual mouse, with all animals independently housed and analyzed as single units. The target sample size (n = 5 per group) was determined by a priori power analysis using GraphPad Prism version 10.1 software. Surgeons and outcome assessors were blinded to group assignments throughout the experiment.

### Appendix A.3. Inclusion and Exclusion Criteria

Inclusion: All mice met the following conditions:

Healthy SD rats (8–10 weeks old, body weight: 200–300 g), 50% male and 50% female.

No pre-operative neurological deficits.

Initial enrollment of 7–8 mice/group (target sample size: n = 5 per group) to account for potential attrition (e.g., intraoperative mortality or technical failure).

### Appendix A.4. Blinding

During the experimental phase, surgeons were blinded to group assignments through the use of randomized coded labels. For data analysis, anonymized samples were independently evaluated by researchers who were unaware of the experimental conditions.
